# Nanophotonics enhanced coverslip for phase imaging in biology

**DOI:** 10.1038/s41377-021-00540-7

**Published:** 2021-05-08

**Authors:** Lukas Wesemann, Jon Rickett, Jingchao Song, Jieqiong Lou, Elizabeth Hinde, Timothy J. Davis, Ann Roberts

**Affiliations:** 1 School of Physics, University of Melbourne, Melbourne, VIC 3010 Australia; 2 ARC Centre of Excellence for Transformative Meta-Optical Systems, School of Physics, University of Melbourne, Melbourne, VIC 3010 Australia

**Keywords:** Imaging and sensing, Nanophotonics and plasmonics

## Abstract

The ability to visualise transparent objects such as live cells is central to understanding biological processes. Here we experimentally demonstrate a novel nanostructured coverslip that converts phase information to high-contrast intensity images. This compact device enables real-time, all-optical generation of pseudo three-dimensional images of phase objects on transmission. We show that by placing unstained human cancer cells on the device, the internal structure within the cells can be clearly seen. Our research demonstrates the significant potential of nanophotonic devices for integration into compact imaging and medical diagnostic devices.

## Abstract (web summary)

The nanophotonics enhanced coverslip (NEC) enables ultra-compact phase imaging of samples placed directly on top of the device. Visualization of artificial phase objects and unstained biological cells is demonstrated.

## Graphical abstract

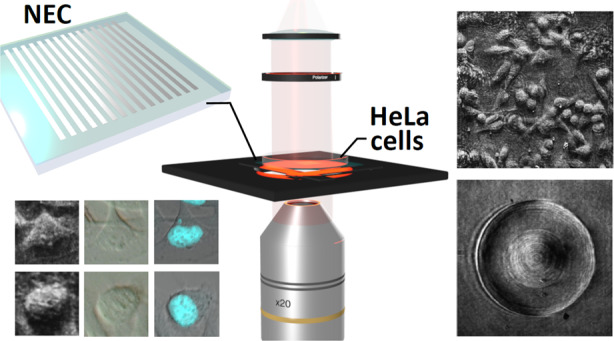

## Introduction

Phase-contrast microscopy has had a profound impact on biology, enabling weakly absorbing microscopic organisms to be observed without staining or fixing^1^. Recently, nanostructured thin-film devices have been developed with the potential to replace the bulky optics used in traditional phase-contrast microscopes. These devices perform mathematical operations on wavefields, such as first- and second-order differentiation^2–4^, and have been used to enhance the edges of features in optical amplitude and phase images^5–13^. Unlike edge-detection that is easily performed digitally, phase visualisation requires processing prior to or at the point of measurement. Here we demonstrate a thin-film device, a nanophotonics enhanced coverslip (NEC), that generates high-contrast images of pure phase objects on transmission. We discuss the underlying principle and demonstrate the device experimentally by imaging the internal structure of human cancer cells (HeLa). Our work highlights the potential of nanophotonic devices in highly compact phase-imaging systems.

The invention of the phase-contrast microscope by Frits Zernike^14^ earned him the Nobel Prize for Physics in 1953. The invention came from Zernike’s analysis of the phase profile of light that he describes as a sum of plane waves travelling in different directions^15^. The absence of intensity contrast results from an ideal convolution of all the plane wave components, such that a perturbation to any one of them disrupts the convolution creating contrast. The same concept underpins X-ray phase-contrast imaging with diffracting crystals that modify the components of the incident wave according to their angles of incidence^16–18^. Similarly, phase contrast arises on wave propagation far from a phase object where the propagating wave components experience different phase shifts and interfere^19^, an effect exploited in the transport of intensity method^20–23^. More generally, the phase-contrast mechanism spatially filters the light in an optical system and can be described by an optical transfer function^24^.

The optical transfer function (OTF), which maps the incident light field to the transmitted light, can be used to understand how a nanophotonic device can create phase contrast^25^. Consider a simple one-dimensional OTF ${\cal{M}}(k_x)$ that depends on the projection *k*_*x*_ of the incident wavevector across the surface of the device. A pure phase wavefield *E*(*x*, *z* = 0) = *e*^*iϕ*(*x*)^ incident on the surface at *z* = 0 is expanded as a sum of plane waves $E(x,0) = {\int} {a(k_x)e^{ik_xx}{\mathrm{d}}k_x}$. The intensity of this wave *I*(*x*,0) = |*e*^*iϕ*(*x*)^|^2^ = 1 shows no contrast. The nanophotonic device modifies the wave in Fourier space according to $E_m(x,0) = {\int} {a(k_x){\cal{M}}(k_x)e^{ik_xx}{\mathrm{d}}k_x}$. Close to *k*_*x*_ = 0 it is always possible to expand the OTF in a power series ${\cal{M}}(k_x) = \mathop {\sum}\nolimits_{n = 0}^\infty {m_nk_x^n}$. The term *m*_0_ multiplies that part of the wave unperturbed by the sample whereas the term *m*_1_ linear in *k*_*x*_ represents the first derivative of the phase profile, ${\mathrm{d}}\left[ {e^{i\phi (x)}} \right]/{\mathrm{d}}x = {\int} {ik_xa(k_x)e^{ik_xx}{\mathrm{d}}k_x = ie^{i\phi (x)}{\mathrm{d}}\phi (x)/{\mathrm{d}}x}$. When *m*_*n*_ = 0 for all *n* ≠ 1 the intensity *I* = |d*ϕ*(*x*)/d*x*|^2^ exhibits phase contrast proportional to phase gradients in the wavefield. In this way, a spatial-frequency filter converts phase differences into intensity contrast. Likewise, higher-order terms in *k*_*x*_ lead to combinations of higher-order phase derivatives that also yield contrast. This is similar to Zernike’s method in which the filtering property is such that *m*_0_ = 1 and $m_n = i = \sqrt { - 1}$ for *n* > 0 that shifts the phase of the diffracted wave by 90°^15^.

## Results

**Fig. 1:**
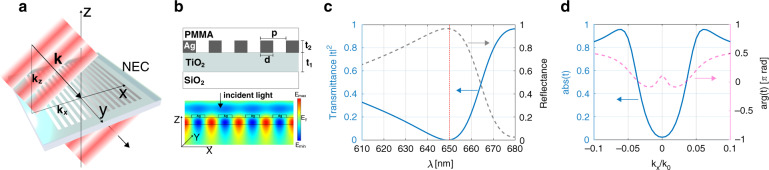
The nanophotonics enhanced coverslip (NEC) and simulations of its performance. **a** A sketch of the NEC showing the geometry and the incident wave vectors. **b** A cross-section used in a FEM simulation and the calculated electric field distribution (ŷ component) for normally incident light at a wavelength *λ* = 650 nm, showing the standing wave induced in the waveguide. The design parameters are *t*_1_ = 100 nm, *t*_2_ = 40 m, *d* = 200 nm and *p* = 400 nm. **c** The calculated frequency response for transmission and reflection of normally incident light identifying the resonance at *λ* = 650 nm. **d** The calculated complex optical transfer function for s-polarised light ${\cal{M}}(k_x)$ at the resonance wavelength *λ* = 650 nm, expressed as a modulation transfer function $\left| {{\cal{M}}(k_x)} \right|$ and a phase transfer function $\arg {\cal{M}}(k_x)$

Our nanophotonic device exploits the contrast-forming properties of spatial-frequency filters to reject the unscattered wave components from a sample but transmit those components with non-zero phase gradients. Sensitivity to propagation direction is achieved with a buried waveguide that samples and interferes each wavefront over a large distance across the surface (Fig. 1a).

Light is coupled into and out of the waveguide by a diffraction grating, composed of metal stripes, where the grating is diffracting into only the higher refractive index of the waveguide layer^26^. Light incident between the stripes is diffracted into three beams—a zero-order diffracted beam (*n* = 0) that propagates in the incident direction and two first-order beams (*n* = ±1) trapped in the waveguide. On reaching the metal stripes, the *n* = ±1 beams reflect with a *π* phase shift and diffract to reform the original zero-order beam. This reformed beam propagates in parallel with the incident light but out of phase, resulting in destructive interference and inhibiting transmission. Changing the incidence angle alters the phase relationship between the beams leading to incomplete cancellation of the transmitted light. In this way, the device blocks the unscattered light from a sample but progressively transmits all other components as their incidence angles, or equivalently their spatial frequencies, increase.

We fabricated the NEC with the geometry in Fig. 1b, using electron beam lithography (see ‘Methods’ section) and characterised its response numerically and experimentally. The grating was overcoated with PMMA to inhibit oxidation and to avoid contamination by the sample. The waveguide thickness is such that it supports only the fundamental waveguide mode. The operation of the device was modelled using the finite element method (FEM) in COMSOL Multiphysics that includes the polarisation dependence. The optical properties for silver were taken from Johnson and Christy^27^ and the refractive index of a TiO_2_ thin-film similar to that used in the waveguide layer was measured experimentally yielding a value *n*_wg_ = 2.25 at *λ* = 633 nm. The calculated electric field distribution for normally incident light polarised parallel to the stripes (Fig. 1b) shows the standing waveguide mode and no transmission. The spectrum (Fig. 1c) has a broad resonance at *λ* = 650 nm where transmission is inhibited. At this wavelength, the OTF (Fig. 1d) also shows that normally incident light is blocked but that the intensity of light transmitted increases with increasing spatial-frequency *k*_*x*_ across the surface for *s*-polarisation, as required for phase-contrast imaging. In addition, the device exhibits a phase transfer function that varies within a limited range of approximately *π*/2, which is important to avoid artefacts in processed images. Here we focus our discussion on *s*-polarised illumination, where the device exhibits stronger angular sensitivity than for *p*-polarisation. High-pass spatial-frequency filtering using this device is also reproduced by the simple model in the Supplementary Information, which supports our understanding.

**Fig. 2:**
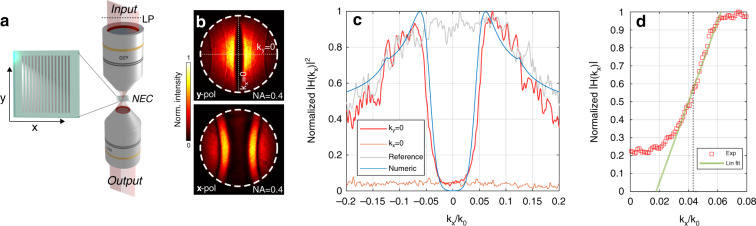
Experimentally measured optical response of the fabricated device. **a** The configuration for measuring the optical transfer function. A linear polariser selects the required polarisation direction relative to the direction of the grating stripes. The output lens produces a Fourier plane image. **b** The experimentally measured normalised two-dimensional modulation transfer function $\left| {{\cal{M}}(k_x,k_y)} \right|^2$ for incident light polarised parallel (*y*-pol) and perpendicular to the silver grating stripes (*x*-pol). The measurement wavelength was *λ* = 637 nm. **c** Profiles along the dashed lines in **b** (red lines) highlighting the suppression of the direct beam. The corresponding simulated profile for *s*-polarisation is given for comparison (blue) with the spatial-frequency content of the incident beam (grey). **d** The normalised modulation transfer function $\left| {{\cal{M}}(k_x,k_y)} \right|$ in the vicinity of *k*_*x*_/*k*_0_ = 0.04. A small tilt in the incident beam relative to the surface normal of the NEC shifts the spatial-frequency origin (dashed line) introducing an asymmetry into the optical transfer function

The modulation transfer function $\left| {{\cal{M}}(k_x,k_y)} \right|^2$ is measured experimentally with the fabricated device in the focal plane between two microscope objectives (Fig. 2a). Fourier images (Fig. 2b) form at the back focal plane of the second objective. We note that the fabricated device was resonant at *λ* = 637 nm, close to the design wavelength of *λ* = 650 nm, the difference related to fabrication errors and differences between published values of refractive index and those obtained with thin-film deposition. The device exhibits high-pass spatial-frequency filtering along *k*_*y*_ = 0, for which the incident light is purely TE (*s*)-polarised. Transmission is suppressed along *k*_*x*_ = 0 (dashed lines in Fig. 2b) as highlighted in Fig. 2c. It should be noted that this implies that the device will not enable phase-visualisation along the *y*-direction. We find good agreement between experiment and numerical simulations with transmission increasing almost parabolically up to *k*_*x*_/*k*_0_ = 0.062, corresponding to an angle of incidence of 3.5°, beyond which transmission drops due to diffraction back into the PMMA layer. The small peaks at *k*_*x*_/*k*_0_ = ±0.13 correspond to a critical diffraction angle where one of the waveguide modes begins to leak into the substrate.

Edge detection of low-contrast features has been demonstrated previously^28^ using a nanophotonic device in the deep red and near infra-red wavelengths that highlight regions where the phase gradients are large. For practical utility in biological imaging, we need to observe gradual phase changes in a wavefield, which can be achieved using the linear region of the optical transfer function (Fig. 2d). The region in which the OTF is near-linear is relatively narrow (approx. 0.03 < *k*_*x*_/*k*_0_ < 0.06). While this limits the range of spatial frequencies that can be filtered in a linear fashion, this does not limit the NA of the objective over which the NEC can be used. Below we demonstrate that this is suitable for visualising micrometre sized phase gradients. We impose an asymmetric OTF by tilting the nanophotonic device relative to the illumination^8,25^ to shift the operation point where the transfer function shows a near-linear dependence, which preferentially transmits the positive spatial frequencies and suppresses the negative frequencies (Fig. 2d).

**Fig. 3:**
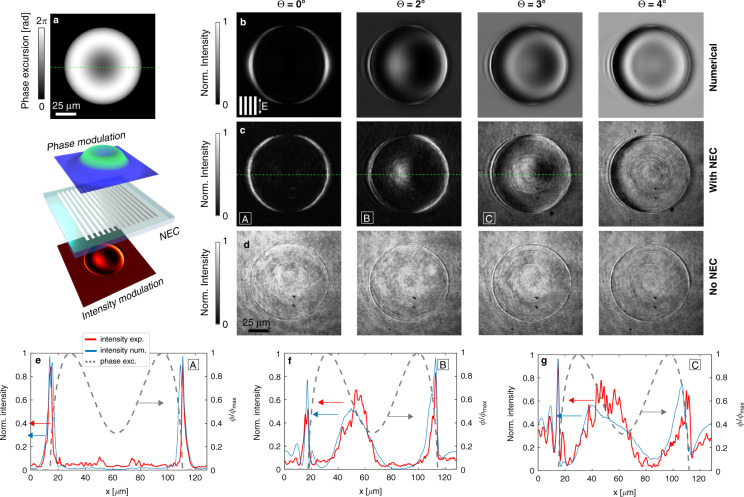
Experimental phase visualisation showing the change in phase contrast with tilt angle of the NEC for incident light polarised along the grating stripes. **a** The phase profile generated by the spatial light modulator, inspired by a magnified model of a red-blood cell, and the conceptual configuration using the NEC. **b**–**d** Four examples of phase imaging each with the incident light tilted at a different angle Θ relative to the NEC surface normal (**b**) calculated images, (**c**) measured images with the NEC, grating orientation and incident polarisation as indicated by inset in **b**. **d** Measurements without the NEC. **e**–**g** Profiles of the intensity along the dashed line through three images (red) as labelled by **e** A, **f** B, and **g** C that show quantitatively the increased transmission associated with phase gradients. Included in the graphs are the phase profile of the red-blood cell model (dashed grey) and the calculated contrast profiles (blue)

We demonstrate this effect with our NEC using the optical configuration discussed in the Supplement. A spatial light modulator (SLM) imposes a computer defined phase-modulation using the (scaled) geometry and optical properties of a red-blood cell^29^ (Fig. 3a). The light field modulated by this phase is passed through the NEC and imaged onto a camera. Figure 3b, c compares a numerical simulation of the expected image (Fig. 3b) with that obtained experimentally (Fig. 3c). The image obtained in the absence of the NEC (Fig. 3d) shows little or no contrast. Transmission of the phase-image through the NEC at normal incidence (Θ = 0° in Fig. 3c) enhances the edges of the phase object where the phase gradients are large. Tilting the NEC between 2° and 4° shows the more subtle phase variations near the centre of the phase object that appear as pseudo three-dimensional relief images similar to conventional bulk-optical phase-visualisation methods. The plots in Fig. 3e–g demonstrate quantitatively the effect of tilting the NEC. The fringes observed at the edges of the ‘sample’ are Fourier filtering artefacts^30^. These artefacts are accurately reproduced in the numerical simulations.

**Fig. 4:**
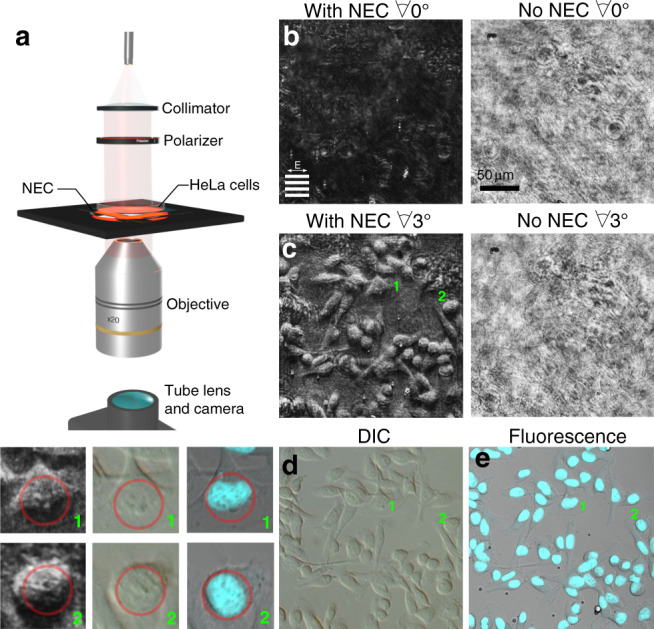
Phase-imaging HeLa cells. **a** The experimental arrangement—637 nm light is collimated and polarised with the electric field parallel to the grating lines before passing through a Petri dish containing fixed HeLa cells above the NEC. On passing through the NEC the light is collected by a ×20 objective and imaged onto a camera. **b**, **c** Images obtained for the same region of cells with and without the NEC at two different tilts with respect to the incident light **b** no tilt Θ = 0°, **c** tilt Θ = 3°. The regions labelled 1 and 2 are magnified in the inset. Grating orientation and incident polarisation as indicated by inset in **b**. **d** Conventional microscope images of the same cells using DIC (left panel) and fluorescence (right panel). The blue regions in the fluorescence image highlight the cell nuclei. Inset: Enlargements of the images obtained by the NEC at Θ = 3° tilt, the DIC and fluorescence microscopes as indicated by the numbers. The circles highlight nucleoli inside the nucleus of each cell

For a practical demonstration, we use the NEC to generate phase-contrast images of biological cells (Fig. 4). Human cancer cells (HeLa) were prepared as described in the ‘Methods’ section with a fluorophore to enable subsequent analysis. The excitation 405 nm and emission 460 nm wavelengths of this fluorophore are sufficiently different from the NEC operation wavelength of 637 nm that the fluorophore does not provide contrast. Images were obtained using the configuration in Fig. 4a to form a collimated light beam that passes through the cells, the NEC and is then imaged into a camera. Figure 4b shows images of the HeLa cells both from unpatterned regions of the substrate and through the NEC with normally incident light. Although some edge features are discernible, the phase variation is too weak to observe much detail. However, a small tilt of the NEC dramatically enhances the phase contrast of the cells (Fig. 4c). For comparison, we also image the same region using the standard phase-contrast technique of differential interference contrast (DIC), shown in the left panel of Fig. 4d. The NEC produces a three-dimensional contrast effect similar to DIC but without the bulk-optical components such as Wollaston prisms required for DIC. The features in our phase images are correlated with the HeLa cell nuclei, as seen by the fluorescence images in Fig. 4d (right panel) where the cell nuclei fluoresce blue. In this configuration, the device acts like a coverslip that automatically provides phase contrast with no additional optics. Moreover, the NEC yields high-resolution images that hint at details of the internal structure of the cells. The insets in Fig. 4 highlight nucleoli inside two of the cell nuclei, features that are invisible in the raw (no NEC) images. A video demonstrating the effect of inserting the NEC into the beam path using the configuration from Fig. 4c to perform phase imaging on HeLa cells is provided in the supplementary information (Supplementary Movie S1).

## Discussion

Although we have demonstrated a simple device that generates phase contrast, it is possible to engineer more complex OTFs with a two-dimensional grating pattern as for example demonstrated theoretically for edge-detection applications in^31^. Additional control of the phase and resonances can be achieved by replacing the simple metal stripes with plasmonically active nanostructures^32^ that individually respond to phase gradients^33^. Resolving the optical phase is essential in many fields, particularly biology, where the inability of detectors to sample phase necessitates the use of additional optics or computational post-processing. The advantage of this structure is that the sample can be placed directly onto it or indeed, the device can be placed at any point between the sample and the detector provided it intercepts all the scattered light. We envisage much greater application of nanophotonics devices, meta-devices and metasurfaces in the near future.

## Methods

### Finite element method modelling

The NEC structure was modelled using the finite element method (FEM) as implemented in COMSOL Multiphysics 5.5 with RF module. The calculation employed Floquet boundary conditions in the transverse *x* and *y* directions and port boundary conditions terminating the model at the upper and lower boundary of the model. Electromagnetic waves are launched into the model via the upper port boundary of the model and the polarisation dependent complex optical transfer function was determined through the *S*-parameters. A tetrahedral mesh with locally increased resolution is used with the maximum element size set to 25 nm. The optical properties for silver were taken from Johnson and Christy^27^ and the refractive index of a TiO_2_ thin-film that we deposited was measured experimentally with a value *n*_wg_ = 2.25 at *λ* = 633 nm.

### Fabrication

A 100-nm-thick layer of TiO_2_ was deposited on top of a 4-inch glass wafer by physical vapour deposition at a deposition rate of 0.5 Å s^−1^ (Intlvac Nanochrome II). The surface pattern was written into a single layer of polymethyl methacrylate resist (PMMA: 280 nm A4, baked at 180 °C for 3 min after deposition) that was spun onto the sample, using an electron beam lithography tool (Vistec EBPG 5000). The sample was developed in a 3:1 mixture of isopropanol: methylisobutyl. Subsequently, a 40-nm-thick layer of silver was deposited on the sample through physical vapour deposition on a 2 nm adhesion layer of chromium. The silver array was embedded in a near-homogeneous environment to protect it from degradation due to exposure to air by spinning a 750 nm thick layer of PMMA onto the sample and baking at 180 °C for 3 min.

### Cell culture, transient transfection, and fixation

Hela cells were grown in DMEM (Lonza) supplemented with 10% bovine growth serum (Gibco), 1× Pen-Strep (Lonza) at 37 °C in 5% CO_2_. Hela cells were plated 24 h before experiments onto 35 mm glass-bottom dishes. Transfected Hela cells were fixed with 4% PFA for 15 min and DNA was stained with 1 μM Hoechst 33342 for 10 min at room temperature.

### Microscope imaging

The setup used for the imaging of human cancer cells via the NEC device was established on a Nikon Ti-80i inverted microscope. Light from a fibre coupled Fabry–Pérot laser diode (Thorlabs S1FC635) with a central wavelength of 637 nm and 1 nm bandwidth (FWHM) was guided to the setup through a single-mode fibre (Thorlabs SM600) where the beam was linearly polarised (Thorlabs LPVIS050-MP2) and collimated using a Nikon LU PLAN Fluor 5×0.3NA objective. The illumination system is mounted on an *xyz*-stage with an integrated rotation module enabling adjustment of the illumination angle. The NEC is placed on the microscope stage and the Petri dish (Fluoro Dish FD35-100) containing the HeLa cells directly on top of it. The collimated light is then directed through the Petri dish and the NEC and the processed image collected by a Nikon LU PLAN 20×0.40NA objective and imaged onto a camera (Andor Zyla 4.2 P). Differential interference contrast (DIC) images for comparison were obtained using an Olympus BX60 microscope using an Olympus PlanN ×20 objective.

## Supplementary information

Supplementary Information

Nanophotonics Enhanced Cover Slip - Phase contrast imaging
