# Supplementary material for: Nanophotonics enhanced coverslip for phase imaging in biology
Source: Light Sci Appl. 2021 May 8;10:98. doi: 10.1038/s41377-021-00540-7 (PMC8106685; doi:10.1038/s41377-021-00540-7)
Supplement: Supplementary file 1 — Supplementary Information [file 41377_2021_540_MOESM1_ESM.pdf]

Supplementary Information for

Nanophotonics enhanced coverslip for phase imaging in biology

Lukas Wesemann[*,1,2], Jon Rickett[1], Jingchao Song[1], Jieqiong Lou[1], Elizabeth Hinde[1], Timothy J. Davis[1], Ann Roberts[*,1,2]

[1]School of Physics, University of Melbourne, Victoria 3010, Australia

[2]ARC Centre of Excellence for Transformative Meta-Optical Systems, School of Physics, University of Melbourne, Victoria 3010, Australia

Contact details

Lukas Wesemann
Lukas.wesemann@unimelb.edu.au
ph: +61 3 8344 8171
School of Physics
The University of Melbourne
Victoria 3010
Australia

Jon Rickett
jrickett@student.unimelb.edu.au

Jingchao Song
jesse.song@unimelb.edu.au

Jieqiong Lou
jieqiong.lou@unimelb.edu.au

Elizabeth Hinde
elizabeth.hinde@unimelb.edu.au

Timothy J. Davis
timd@unimelb.edu.au

Ann Roberts
ann.roberts@unimelb.edu.au
ph:+61 3 8344 5038
School of Physics
The University of Melbourne
Victoria 3010
Australia

Simple model of nanophotonic device

Although it is possible to write down a full series solution of Maxwell's equations to model the nanophotonic device, the physics that underpins its operational principles becomes obscured in the mathematical detail. Instead, here we develop an approximate analytical model, which is not used for accurate prediction of the spatial filtering properties but which highlights the basic principles of the device. There are two main factors that affect the device performance, both of which are related to the grating. Since the in-plane wavenumber is continuous across all layers in the device we have $n_\mathrm{i} \sin\theta_\mathrm{i} = n_\mathrm{p} \sin\theta_\mathrm{p} = n_\mathrm{w} \sin\theta_\mathrm{w}$ where n_i and θ_i are the refractive index and angle of the incident wave, n_p and θ_p are the corresponding parameters for the PMMA layer and n_w and θ_w those for the waveguide. This equation is Snell's law.

The grating has refractive index n_p on top (PMMA layer) and n_w beneath (TiO_2 waveguide layer) and a periodicity d. The grating equation for waves diffracted back into the PMMA layer at angle θ_np is given by $n_\mathrm{p} \sin\theta_\mathrm{np} = n_\mathrm{p} \sin\theta_\mathrm{p} + n\lambda/d$. For the waveguide, the corresponding formula for transmission into the waveguide is $n_\mathrm{w} \sin\theta_\mathrm{nw} = n_\mathrm{p} \sin\theta_\mathrm{p} + n\lambda/d$ where n is an integer representing the grating order. From Snell's law, these formulas can be rewritten in terms of the wave incident on the device so that

$$n_\mathrm{p} \sin\theta_\mathrm{np} = n_\mathrm{i} \sin\theta_\mathrm{i} + n\lambda/d \tag{1}$$

and

$$n_\mathrm{w} \sin\theta_\mathrm{nw} = n_\mathrm{i} \sin\theta_\mathrm{i} + n\lambda/d \tag{2}$$

The waveguide is excited by waves incident on the gaps between each silver strip of the grating, resulting in a large number of waves propagating along the waveguide as well as a wave continuing to propagate in the incident direction. A crude formula for amplitude of the nth diffraction order for a grating of this type is [S1]

$$E_n \approx (E_0/2)\mathrm{sinc}(n/2) \tag{3}$$

with E_0 the incident wave amplitude and $\mathrm{sinc}(x) = \sin(\pi x)/\pi x$. These waves combine and interfere constructively or destructively depending on their wavelength relative to that of the grating period. Thus we can sum all the waves from each grating gap that arrive at the central strip at $x = 0$ to write down an expression for the amplitude of the nth diffracted order:

$$E_n = (E_0/2)\mathrm{sinc}(n/2)\left(e^{ik_x d/2}\sum_{m=1}^{\infty} e^{ik_x(md)-\gamma(md)} + e^{-ik_x d/2}\sum_{m=1}^{\infty} e^{-ik_x(md)-\gamma(md)}\right) \tag{4}$$

where the first term is all the waves from the left of the central strip propagating to the right, and the second term is all the waves to the right propagating to the left. The phenomenological factor γ accounts for intensity loss due to light scattering out of the waveguide. These sums are easily evaluated giving

$$\begin{aligned} E_n &= (E_0/2)\mathrm{sinc}(n/2)\left(\frac{e^{i3k_x d/2-\gamma d}}{1-e^{ik_x d-\gamma d}} + \frac{e^{-i3k_x d/2-\gamma d}}{1-e^{-ik_x d-\gamma d}}\right) \\ &= E_0\mathrm{sinc}(n/2)e^{-\gamma d}\left(\frac{\cos 3k_x d/2 - e^{-\gamma d}\cos k_x d/2}{1+e^{-2\gamma d}-2e^{-\gamma d}\cos k_x d}\right) \end{aligned} \tag{5}$$

These waves also diffract from the periodic array of metal stripes, but do so in reflection and create another wave now propagating in the original incidence direction. The reflection of waves from a metal surface results in a π phase shift. Thus the electric field of the waves transmitted through the device is a linear combination of the original zeroth-order diffracted wave and the diffracted waveguide waves

$$E_t/E_0 = \mathrm{sinc}(0)/2 - \mathrm{sinc}^2(1/2)e^{-\gamma d}\left(\frac{\cos 3k_x d/2 - e^{-\gamma d}\cos k_x d/2}{1+e^{-2\gamma d}-2e^{-\gamma d}\cos k_x d}\right) \tag{6}$$

The in-plane wave number is $k_x = n_{\mathrm{w}} k \sin\theta_{\mathrm{nw}} = n_{\mathrm{i}} k \sin\theta_{\mathrm{i}} + n 2\pi/d$ with $k = 2\pi/\lambda$ the free-space wavenumber.

The second factor that comes into play is when the grating becomes diffracting in the PMMA layer. This occurs when $|\sin\theta_{\mathrm{np}}| = |n_{\mathrm{i}}\sin\theta_{\mathrm{i}} + n\lambda/d|/n_{\mathrm{p}} < 1$. For the first diffracting orders $n = \pm 1$, diffraction into the PMMA layer occurs when $|k_x/k| > 0.093$. If

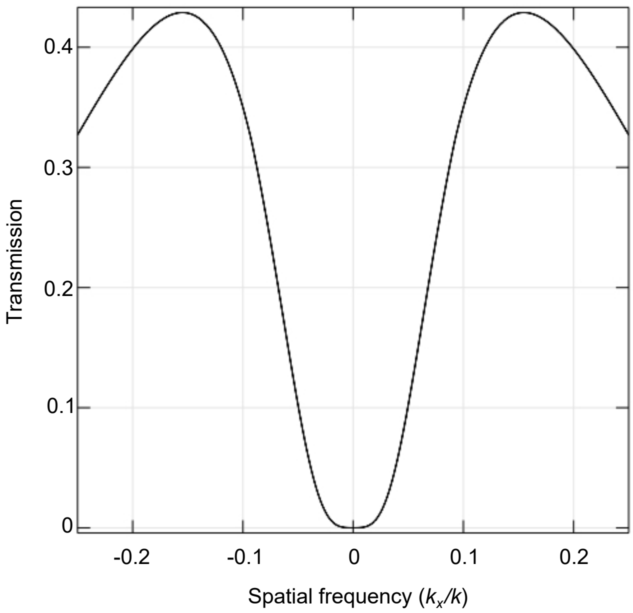

FIG. S1: The transmission from the device based on the diffraction model of the device including a factor accounting for energy loss due to diffraction in the PMMA layer.

we assume the strength of the diffraction varies as $\cos^2\theta_{np}$, which represents the projection of the intensity out of the grating, then the transmission will roll-off for larger spatial frequencies by a factor approximately $1 - \cos^2\theta_{\mathrm{np}} = \sin^2\theta_{\mathrm{np}}$.

The transmission $|E_t/E_0|^2 \sin^2\theta_{\mathrm{np}}$ is plotted as a function of wave number, or spatial frequency, $k_x/k = \sin\theta_{\mathrm{i}}$ with $n_{\mathrm{i}} = 1$ in Fig. S1. For this calculation $\gamma = 0.0015$ per nm and we include the extra factor only when $\sin^2\theta_{\mathrm{np}} < 1$. The refractive index of the upper (PMMA) layer used in the calculation was $n_{\mathrm{i}} = 1.5$ and that of the waveguide $n_{\mathrm{w}} = 2.25$. Although a very crude analysis, this model captures the main features of the optical transfer function of our device. Additionally we would expect the PMMA layer to have Fabry-Perot resonances that will have some influence on the result. We do not model these effects here.

Optical Configuration for SLM Experiments

The optical configuration for the experimental tests using the spatial light modulator is shown in the setup shown in Figure S2. A reflective spatial light modulator (HOLEYE Holoeye Pluto2 VIS014) imposes a computer defined phase-modulation on a wavefield. Collimated light from a fiber coupled Fabry-Perot laser diode (Thorlabs S1FC635) operating at

a fixed wavelength of $\lambda = 637$ nm with a bandwidth of 1 nm (FWHM) is linearly polarised along the operational direction of the SLM. The reflected field is then demagnified through a telescope consisting of a $f = 150$ mm lens (Thorlabs-LA1433-A) (L1) and a micropscope objective (Nikon UPlanFl 20x 0.5NA) (MO2) and the phase-image projected onto the NEC sample. The transmitted image is collected using a microscope objective (Nikon LU Plan 50x 0.55NA) (MO3), and through a $f = 50$ mm lens (Thorlabs LA1131-A) (L3) projected onto a camera (Thorlabs DCC1545M).

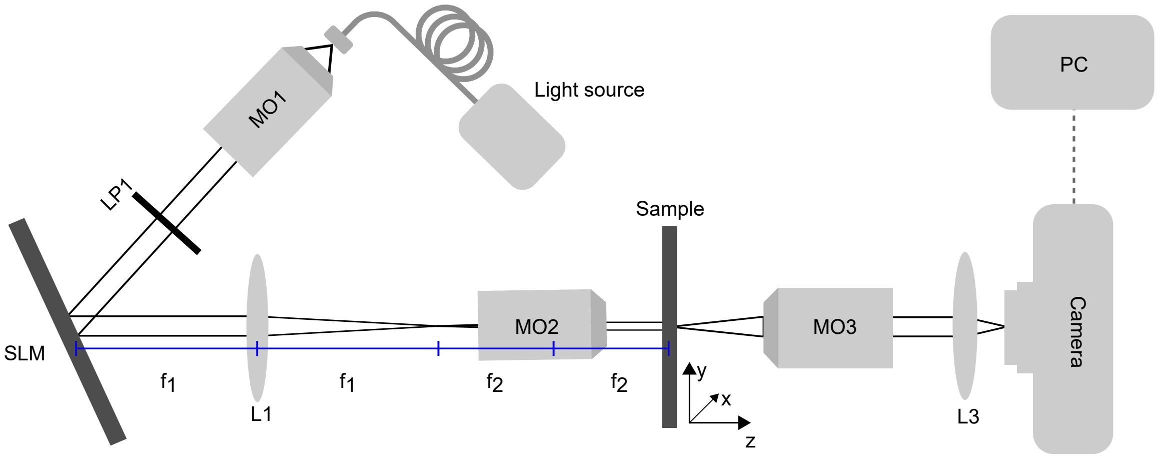

FIG. S2: Experimental setup used for the detection of phase gradients generated through a spatial light modulator (SLM). The generated phase images are demagnified via a telescope (L1, MO2). The NEC device is inserted in the sample-position and the transmitted image collected by a microscope objective (MO3) before it is imaged onto a camera using a lens (L3). The NEC is tilted by small angles along the y-axis in order to offset the optical transfer function.

Supplement References

[S1] Harvey, J. E. & Pfisterer, R. N. Understanding diffraction grating behavior: including conical diffraction and Rayleigh anomalies from transmission gratings, *Optical Engineering* **58**, 1 – 21 (2019)

Supplementary Files

- **Movie S1:** Phase imaging of HeLa cells using the nanophotonics enhanced coverslip. *The movie highlights the performance of the device. The tilt angle of the illumination for this video was set to 3 degrees to introduce an asymmetry into the optical transfer function which produces the pseudo 3D effect. The incident light was polarized along the grating lines.*
